# Shape variation and modularity of skull and teeth in domesticated horses and wild equids

**DOI:** 10.1186/s12983-018-0258-9

**Published:** 2018-04-19

**Authors:** Laura Heck, Laura A. B. Wilson, Allowen Evin, Madlen Stange, Marcelo R. Sánchez-Villagra

**Affiliations:** 1 Palaeontological Institute and Museum, University of Zurich, 8006 Zurich, Switzerland; 2 Palaeontology, Geobiology and Earth Archives Research Centre, School of Biological, Earth and Environmental Sciences, University of New South Wales, Sydney, NSW 2052 Australia; 3 Institut des Sciences de l’Evolution – Montpellier, CNRS UMR 5554, Université de Montpellier, IRD, EPHE, 2 place Eugène Bataillon, 34095 Montpellier, France; 4 Department of Archaeology, Classics and Egyptology, University of Liverpool, Liverpool, UK

**Keywords:** Domestication, Disparity, Modularity, Geometric morphometrics, Cranium

## Abstract

**Background:**

In horses, the morphological changes induced by the process of domestication are reportedly less pronounced than in other species, such as dogs or pigs – although the horses’ disparity has rarely been empirically tested. We investigated shape differences and modularity of domesticated horses, Przewalski’s horses, donkeys and zebras. Mandibular and tooth shape have been shown to be valuable features for differentiating wild and domesticated forms in some mammals.

**Results:**

Both mandible and teeth, show a pattern of shape space occupation analogous to that of the cranium, with domesticated horses occupying a similar extension in shape space to that of wild equids. Only cranial shape data exhibit a tendency to separate domesticated horses and Przewalski’s horses from donkeys and zebras. Maximum likelihood model-based tests confirm the horse cranium is composed of six developmental modules, as reported for placental mammals in general. The magnitude of integration in domesticated horse skull was lower than in wild equids across all six cranial modules, and lower values of integration were associated with higher disparity values across all modules.

**Conclusion:**

This is the first study that combines different skeletal features for the description and comparison of shape changes in all living equid groups using geometric morphometrics. We support Darwin’s hypothesis that the shape variation in the skull of domesticated horses is similar to the shape variation of all wild equid species existing today. Lower magnitudes of module integration are recovered in domesticated horses compared to their wild relatives.

**Electronic supplementary material:**

The online version of this article (10.1186/s12983-018-0258-9) contains supplementary material, which is available to authorized users.

## Background

After being on the verge of extinction, domestication made horses one of today’s most common large animal species [1]. All living species of equids belong to the genus *Equus,* which is divided into the caballine taxa, including domesticated horses (H) and Przewalski’s horses (P), and non-caballine taxa, comprising the different donkey (D) and zebra species (Z). Within the caballine taxa, the Przewalski’s horses likely represent the sister-taxon to the extinct wild ancestor of domesticated horses [2, 3]. Since the early domestication of horses, reproductive isolation promoted divergence by genetic drift and natural selection. Later on, extensive selective breeding to meet human needs for certain behavioural or physiological traits resulted in a wide range of morphological variation [4–6]. Horses, like other domesticated species, have been shaped into diverse morphological types through artificial selection to fit specific functions, such as agricultural work, racing, or leisure. Four traditional body types are recognized: draft horses, medium horses, light horses, and ponies [5, 6]. Horse disparity was already acknowledged by Charles Darwin, who noted that its intraspecific disparity is larger than the interspecific disparity of equids in general [7]. Darwin proposed that great differences among horse breeds can be found in the skull. Based on its complexity in form and origin, as well as its relation to important vital functions, the skull is the most commonly used marker of morphological variation [8]. The increase in skull shape variation following domestication has been found in different domesticated species such as dogs [9], cattle [10], and pigeons [11], where it has been measured using geometric morphometric methods, and quantified through comparisons of variance in shape space. In addition, potential shape changes in teeth are commonly used in zooarchaeological studies to determine the time and location of domestication [12–16]. Previous studies on skull and tooth morphology and morphometrics show the existence of intraspecific as well as interspecific shape variation in subsets of the equid clade (Table 1). However, the patterning and magnitude of variation in skull shape or tooth shape across all extant equids has so far not been examined. In order to quantify the shape variation in extant equids and to investigate the impact of domestication, we first compare domesticated horses represented by 38 different breeds and encompassing the whole size range of the species, to the extant zebra and donkey species, as well as to the Przewalski’s horse (Table 2) using two- and three-dimensional geometric morphometrics to explore cranial and mandible (3D), and teeth (2D) morphometrical variation.

**Table 1:** Overview of previous literature on skull and/or tooth morphology and morphometrics in extant equids

Author	Species and/or breeds	Body part	Method	Summary
Bennett (1980) [38]	*Equus andium, E. asinus, E. burchelli, E. caballus* (including *E. caballus alaskae,* originally named *E. niobrarensis alaskae* by Hay, 1915)*, E. calobatus, E. conversidens, E. francisi, E. grevyi, E. hatcheri, E. hemionus, E. kiang, E. onager, E. occidentalis, E. quagga, E. scotti, E. zebra,* and *Dinohippus*	Skull & teeth	Descriptive morphology	Living species of *Equus* can be differentiated by a number of morphological characters
Seetah et al. (2016) [14]	Icelandic, Thoroughbred, Przewalski’s horses, and potentially *E. ferus*	Teeth	2D geometric morphometrics	Tooth shape of horses largely resembles those of Pleistocene and recent wild horses until the onset of modern breeds
Seetah et al. (2014) [48]	Icelandic and Thoroughbred horses	Teeth	2D geometric morphometrics	Significant differences between the two horse breeds in tooth shape
Evans & McGreevy (2006) [63]	Thoroughbreds, Standardbreds, Ponies, Arabs, Anglo-Arabs, Quarter horse, Warmblood, and Appaloosa	Skull	Classic morphometrics	No overall shape differences exist but modular differences (nasal vs. cranial)
Zhu et al. (2014) [64]	*E. asinus* compared to ponies from Jie (1995) and Evans & McGreevy (2006)	Skull	Classic morphometrics	Supports the two modules from Evans & McGreevy (2006) and shows that donkeys have a longer nasal part
Hanot et al. (2017) [39]	Domestic horses (*E. caballus*) of various breeds (i.e. racehorses, draft horses, Shetland ponies, Icelandic ponies, Camargue horse, Pottok, Konik), Przewalski’s horses (*E. przewalskii*), domestic donkeys (*E. asinus asinus*) and wild asses (*E. a. africanus*), mules (*E.asinus x E. caballus*) and hinnies (*E. caballus x E. asinus*)	Skull & skeleton	3D geometric morphometrics	Occipital part of the skull is especially discriminant among species and it is possible to identify domesticated equids from archaeological sites
Cucchi et al. (2017) [16]	*E. ferus caballus, E. f. przewalskii, E. africanus somaliensis, E. a. asinus, E. kiang, E. hemionus hemionus, E. h. khur, E. h. kulan, E. grevyi, E. zebra hartmannae, E. quagga quagga, E. q. burchelli,* and hybrids (donkey*horse)	Teeth	2D geometric morphometrics	Enamel folding is a good phylogenetic marker; strong taxonomic pattern is visible in enamel folding
Eisenmann & Baylac (2000) [65]	*E. grevyi, E. burchelli boehmi, E. zebra zebra, E. asinus, E. h. kulan, E. przewalskii,* and *E. caballus*	Skull	Classic morphometrics	Domestic horses and Przewalski’s horses can be differentiated

**Table 2:** Number of individuals in each group (domesticated horses (H), Przewalski’s horses (P), donkeys (D), and zebras (Z)) on each cranium, mandible, and tooth; sample of domesticated horses is present by breed

Group	Breed	Cranium	Mandible	U3M	U2P	L3M	L2P
Domesticated horses (H)	Ancient breed	2	1	4	3	1	1
	Anglo-Norman	2	2	1	2	2	2
	Arab	7	8	6	7	4	2
	Birkenfelder	1	1	1	0	0	0
	Belgian Draft	11	10	9	8	6	9
	Bosnian Pony	1	0	0	0	0	0
	Clydesdale	3	3	3	3	3	2
	Exmoor Pony	1	1	1	1	1	1
	Falabella	1	0	0	0	0	0
	Galician Farm Horse	3	3	2	3	2	2
	Grisons (Graubünden)	3	3	3	3	3	3
	German Riding Pony	2	2	1	2	0	2
	Hannoverian	2	3	2	3	1	1
	Hackney	2	2	2	2	1	1
	Holstein	1	1	1	1	1	0
	Hungarian	3	3	3	3	1	3
	Huzule	3	2	2	2	2	1
	Icelandic Horse	16	18	17	18	12	16
	Indian Pony	2	1	2	2	1	1
	Kladrubian	10	10	10	10	9	6
	Konik	1	1	0	1	0	0
	Kosarian	1	1	1	1	0	1
	Lipizzan	2	2	0	2	0	2
	Mongolian	3	3	3	1	2	1
	Norik	2	2	2	1	1	0
	Oldenburgian	1	1	1	1	1	1
	Pinzgau	18	17	16	17	15	9
	Polish Farm Horse	1	1	1	1	1	1
	Scottish Pony	2	1	1	1	0	0
	Seneca Sarajevo	1	0	1	1	0	0
	Shetland Pony	6	6	5	5	4	5
	Shire	1	1	1	1	1	0
	Styrian	1	1	1	1	1	0
	Suffolk	2	2	1	2	1	1
	English Thoroughbred	6	7	6	7	6	5
	Togo Pony	0	5	4	4	2	3
	Trakehner	3	4	4	4	3	4
	Welsh	6	12	4	6	4	7
Subtotal		133	141	122	130	92	93
Donkeys (D)		31	33	25	24	20	18
Zebras (Z)		47	48	42	41	29	28
Przewalski’s Horses (P)		5	2	3	3	3	2
	Total	216	224	192	198	144	141

As a second part of this study, we examine modularity of the equid skull. The concept of modularity [17, 18] has attracted much attention in recent years (e.g. [19–26]), having emerged as a quantitative framework for exploring questions relating to facilitation and constraint in morphological evolution, with the goal of understanding how (and by how much) the direction in which variation is generated is biased [27–29]. Many studies have quantified patterns of modularity in the cranium using inter-trait correlations extracted from geometric morphometric data (see [30] and references therein) and, taken together, their results have supported a common pattern in therian mammals, with some variability in the magnitude of integration among species (e.g. [20, 31]). In contrast, comparatively little is known about the lability of modular patterning and integration magnitudes on relatively short time scales and under selective breeding regimes, although changes in magnitude, rather than patterning, have been implicated as the target for selection [32]. Providing examples of selective breeding for features to suit human activities, the study of domestication events offers an opportunity to empirically examine the role of modularity and integration in the generation of cranial disparity over short evolutionary timescales. A modular structure of the skull is expected to be uncovered for horses, as has been found across a wide spectrum of mammals (e.g. [31]), and we assess the fit of our shape data to functional and developmental hypotheses for modular patterning [33] that have been previously tested in the mammalian cranium. According to Darwin’s hypothesis, domesticated horses should show more variation in shape than the wild equid species. If this hypothesis is supported, then we should find differences in integration and disparity measures for cranial modules between wild equids and domesticated horses. To do so, we assess whether cranial modules display a) higher or lower magnitudes of integration and b) high or low disparity for domesticated horses and wild equids, and c) we investigate whether there is a relationship between module integration/disparity and regions of the cranium showing most variability in shape among domesticated horses.

Our aim is to characterize and quantify the patterning and magnitude of shape variation in the skulls and teeth of domesticated horses compared to the wild species of *Equus*. We use geometric morphometric methods to: a) test Darwin’s hypothesis that the magnitude of intraspecific disparity in horses is larger than the interspecific disparity in equids, b) examine the extent to which domestication influenced tooth shape in equids, c) investigate whether the patterning of shape variation in horse skulls reflects a modular structure, specifically identifying the model best supported for the landmark data by evaluating four modular hypotheses that reflect developmental and functional trait interactions in the cranium, and d) quantify differences in the magnitude of modularity and integration between domesticated horses and wild equids.

Given the well-documented palaeontological record of horses [34], these animals offer the possibility to compare diversification in macroevolutionary and microevolutionary scale like few others. In fact, previous classic studies by Radinsky [35, 36] investigated some of the cranial transformations with morphometric approaches typical of that time. Our study expands the studies of the extant species using newer morphometric approaches and provides the basis for future works comparing also the fossil record of the clade.

## Results

### Shape variation

**Fig. 1:**
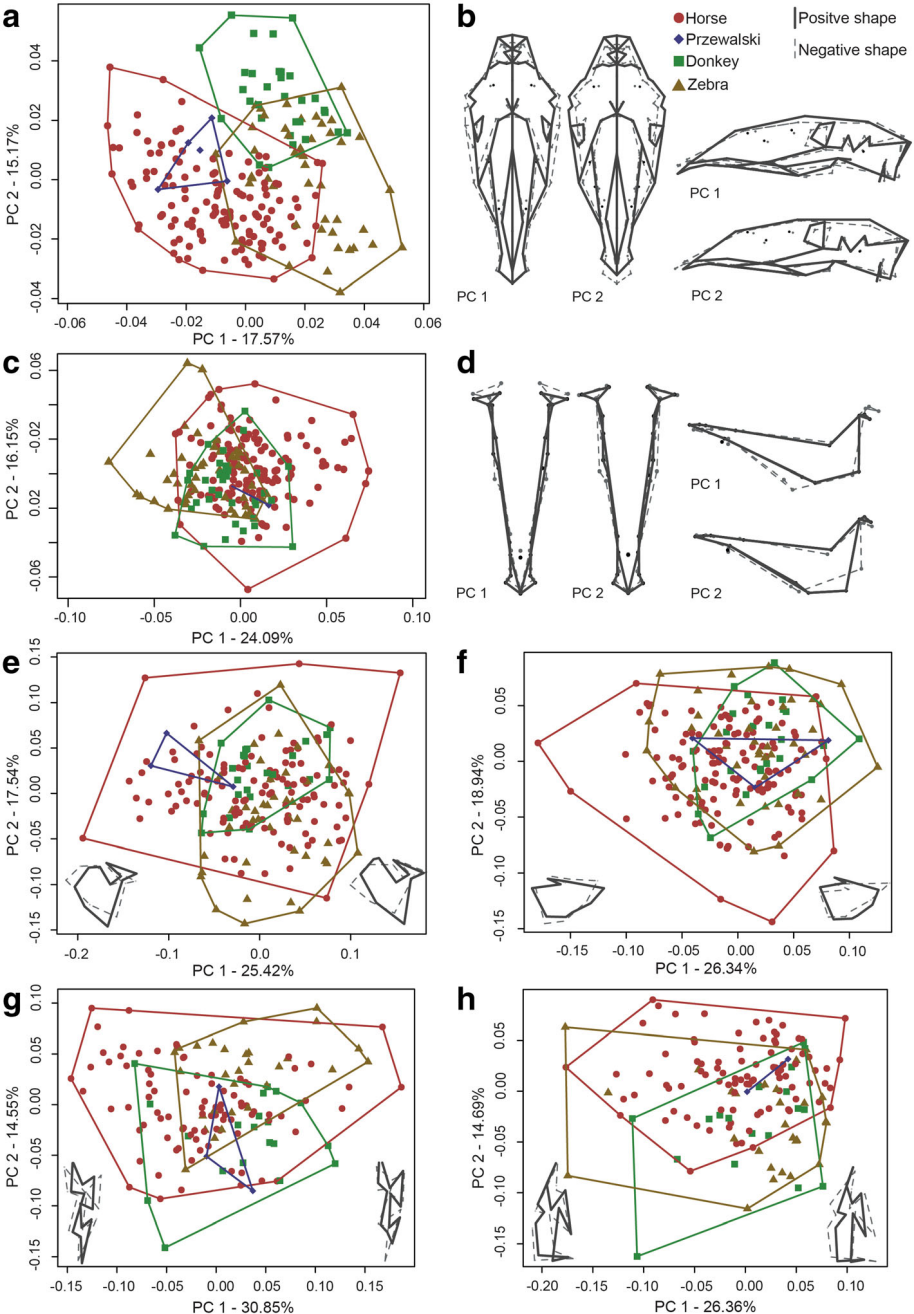
Principal component analysis of **a** the cranial landmark data of 216 adult equid specimens: zebras (*n* = 47), donkeys (*n* = 31), Przewalski’s horses (*n* = 5), and domesticated horses (*n* = 133), **b** Shape changes in dorsal and lateral view for PC 1 and PC 2 with black lines presenting positive shape and dotted, grey lines presenting negative shape, **c** the mandible landmark data of 224 adult equid specimens: zebras (*n* = 48), donkeys (*n* = 33), Przewalski’s horses (*n* = 2), and domesticated horses (*n* = 141), **d** Shape changes in dorsal and lateral view for PC 1 and PC 2 with black lines presenting positive shape and dotted, grey lines presenting negative shape, **e** of the upper 3rd molar landmark data of 225 adult equid specimens: zebras (*n* = 42), donkeys (*n* = 25), Przewalski’s horses (*n* = 3), and domesticated horses (*n* = 122), **f** the upper 2rd premolar landmark data of 225 adult equid specimens: zebras (*n* = 42), donkeys (*n* = 25), Przewalski’s horses (*n* = 3), and domesticated horses (*n* = 122), **g** of the lower 3rd molar landmark data of 225 adult equid specimens: zebras (*n* = 29), donkeys (*n* = 20), Przewalski’s horses (*n* = 3), and domesticated horses (*n* = 92), **h** of the lower 2rd premolar landmark data of 225 adult equid specimens: zebras (*n* = 29), donkeys (*n* = 20), Przewalski’s horses (*n* = 3), and domesticated horses (*n* = 92); Symbols are circles: domesticated horses, diamonds: Przewalski’s, triangles: zebras, and squares: donkeys

In the cranial symmetrised shape data, the first three principal components (PCs) account for 43.1% of the total shape variation in the cranium (Fig. 1a). PC 1 (17.6%) tends to separate the caballine equids (H, P) from the non-caballine equids (D, Z). The shape change along PC 1 from negative to positive is dominated by a narrowing and straightening of the skull in combination with an elongation of the occipital-parietal region, represented by the cranial vault (Fig. 1b). PC 2 accounts for 15.2% of the overall observed variation, and is characterized by a general broadening of the skull in combination with an elongation of the occipital-parietal region (cranial vault module) and a shortening of the nasal region (anterior oral-nasal module). Because of the large number of landmarks compare to the relatively small number of specimens we applied a dimensionality reduction of the datasets by selecting the first PCs for all further analyses following Evin et al. [13] (mevolCVP function) that also takes into account unbalanced sample size between groups. The results of the mevolCVP function suggested the reduction of the dataset to the first three PCs in all further analyses for the cranial data. Significant differences among the four groups (Procrustes ANOVA *p* < 0.001, F = 35.578, based on the 6 first PCs) allowed us to perform a canonical variance analysis (CVA) with a-priori defined groups (H, D, P, Z) resulting in an overall classification accuracy of 78.4% (Confidence interval CI: 60%–95%) when the four groups are analysed, and 98.2% (CI: 96.8%–100%, based on the 17 first PCs, Procrustes ANOVA *p* < 0.001, F = 32.723) when the Przewalski horse specimens are excluded. In this later analysis, both domestic horses and donkeys could be correctly assigned to their respective groups in 100% of the cases. Zebras were assigned correctly for 95.7% of cases and the remaining 4.3% were grouped within the donkeys. Predictive discriminant analyses detect cranial shape proximities of the five Przewalski’s horses with the domestic horses (100% probabilities of identification). Domesticated and Przewalski’s horses are most distinct from donkeys and zebras in Procrustes shape space, as measured by Mahalanobis distance (Table 3). Horses occupy a larger Procrustes shape space (53.12%) as determined by Foote’s partial disparity, than zebras (26.22%) and donkeys (18.34%). The Przewalski’s horses occupy only 2.26% of the overall shape space. The overall Procrustes variance for the cranium is 0.0023.

**Table 3:** MAHALANOBIS DISTANCE

	Cranium			Mandible		
	D	H	P	D	H	P
H	5.67			10.08		
P	5.27	1.04		6.81	2.66	
Z	2.32	6.37	6.31	5.6	9.55	7.81
	Cranium 3M (U3M)			Cranium 2P (U2P)		
	D	H	P	D	H	P
H	1.77			2.56		
P	2.61	1.95		2.82	1.61	
Z	1.83	1.58	2.79	1.85	2.52	3.13
	Mandible 3M (L3M)			Mandible 2P (L2P)		
	D	H	P	D	H	P
H	2.23			2.16		
P	2.33	1.84		2.42	0.96	
Z	2.79	2.02	3.37	1.27	2.32	2.51

The first four PCs of the mandible shape data account for 64.1% of the total shape variation in the mandible. In contrast to the cranium, none of the PCs shows separation between any of the four groups. Specimens of all groups largely overlap in PC shape space (Fig. 1c and d), therefore we do not discuss this further. Due to significant results of the Procrustes ANOVA (*p* < 0.001, F = 14.4, 3 first PCs), we computed a CVA with the same a-priori groups used in the cranium. The overall classification accuracy was low when the four groups were analysed (H, P, D, Z) 38.4% (CI: 12.5%–87.5%), while a classification accuracy of 87% (CI: 82.4%–91.2%, 17 first PCs, F = 15.681, *p* < 0.001) was reached when the two Prezwalski horses were excluded. In this later analysis, 88.2% of the donkeys, 87.9% of the horses and 89.6% of the zebras were correctly classified. The two Przewalski’s specimens were both classified as horses with probabilities of 100% and 72%. Donkeys, zebras, and Przewalski’s horses are similarly spaced from each other (Mahalanobis distance, Table 3). Mandible Procrustes shape space occupation is very similar to the cranial shape space with horses dominating the shape space (Foote’s partial disparity 58.98%). Zebras occupy the second largest shape space with 23.69%, followed by donkeys (16.59%) and Przewalski’s horses (0.71%). The Procrustes variance of the mandible (0.0024) is slightly higher than in the cranium.

In the teeth of the upper tooth row, the first four PCs of the third molar (U3M) account for 66.6%, and of the second premolar (U2P) for 70.1% of the shape variation. In the lower tooth row, only the first three PCs are each above 10% and account for 55.8% in the third molar (L3M) and 51.5% in the second premolar (L2P). None of the PCs show separation of the four groups from each other for any of the teeth, with specimens of all groups largely overlapping in PC shape space (Fig. 1e-h). The four groups differ in the shape of their four teeth (U3M: *p* < 0.001, F = 2.8609, 2 first PCs; U2P: *p* < 0.001, F = 9.0841, 4 first PCs; L3M: *p* < 0.001, F = 6.1919, 4 first PCs; L2P: *p* < 0.001, F = 4.4724, 7 first PCs). We computed CVAs with the same a-priori groups (H, P, D, Z) before removing the smallest group of Przewalski horse, like for the cranium and mandible analyses. The overall classification accuracy was similar among all teeth with 41.3% (CI: 24.6%–66.7%, 6 PCs) for U3M, 39.4% (CI: 16.7%–66.7%, 2 PCs) for U2P, 35% (CI: 8.3%–58.3%, 4 PCs) for L3M, and 34.5% (CI: 11.9%–62.5%, 7 PCs) for L2P. For the later comparison excluding the Przewalski’s specimens, overall classification accuracy was similar among all teeth: 63.9% (CI: 56%–70.7%, 11 PCs) for U3M, 76.4% (CI: 69.4%–83.3%, 12 PCs) for U2P, 75% (CI: 66.6%–81.7%, 8 PCs) for L3M and 70.9% (CI: 63%–79.7%, 9 PCs) for L2P. The Przewalski’s specimens show close shape proximities with horses for L2P (two specimens identified to horses with probabilities of 100% and 96.7%) and U2P (three specimens with probabilities between 91.7% and 95.8%), while for L3M two of the three specimens were closer to donkeys (65.8% and 100%) and the latest to horse (100%), and for U3M for which two specimens were identified as close to horses (100% and 52.9%) and one to donkey (100%).

All analysed teeth (L2P, U2P, L3M, and U3M) have a similar partial disparity as all other analysed features: horses showing the highest partial disparity followed by zebras, donkeys, and Przewalski’s horses. However, the overall disparity (Procrustes variance) differs between the teeth, with cranial P2 showing the smallest variance (0.008) while all other teeth exhibit a total variance around 0.013. The Mahalanobis distances among the groups calculated for each tooth separately are similar for cranial and mandibular P2, and cranial and mandibular M2. Przewalski’s horses and zebras are in all instances most disparate. The P2 is most similar between horses and Przewalski’s horses, the cranial M3 is most similar between zebras and horses and the mandibular M3 is most similar between Przewalski’s horses and horses followed by the zebra (Table 3).

### Modularity

**Fig. 2:**
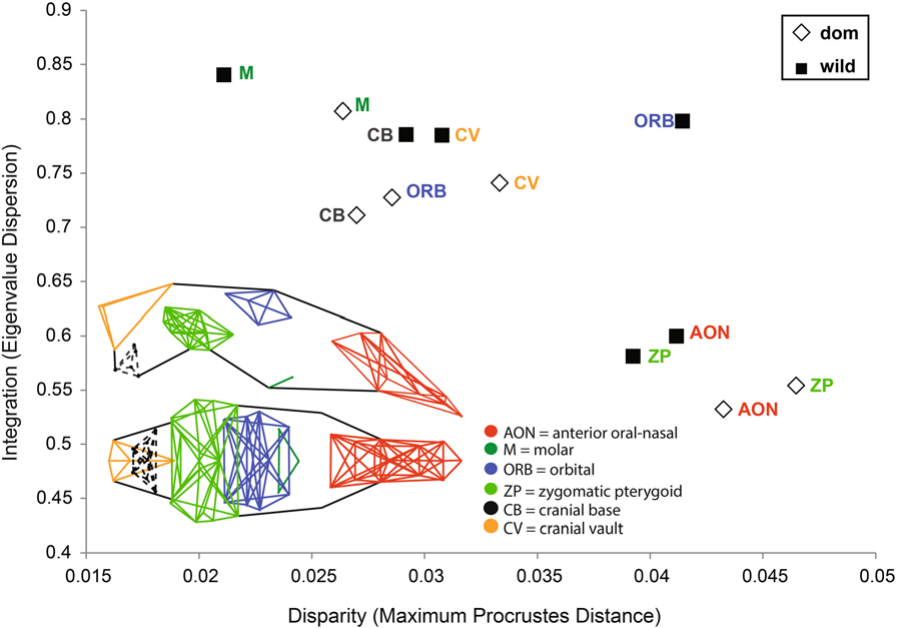
**a** Scatterplot of calculated values for disparity (x-axis) and integration (y-axis) for all modules and wild horses and domesticated equids separately; **b** Six cranial modules in a schematic horse skull (black outline) after Goswami [20] shown in lateral and dorsal perspective; Modules are: anterior oral-nasal (AON, red), molar (M, dark green), orbital (ORB, dark blue), zygomatic pterygoid (ZP, light green), cranial base (CB, orange), and cranial vault (CV, black dashed)

Results from EMMLi indicated that, of the models tested here, the best supported model for modularity was Goswami’s mammalian module hypothesis [20] with separate within-module integration and separate between-module integration (model 2d, Additional file 1: Table S1). This model had the lowest Akaike Information Criterion (AICc) value of − 969.34 and a maximum likelihood of 507.97 (Additional file 1: Table S1). Goswami’s mammalian module hypothesis contains six modules, these are anterior oral-nasal (AON), cranial base (CB), cranial vault (CV), orbit (ORB), molar (MR), and zygomatic-pterygoid (ZP) (Fig. 2, [20]). Disparity and integration values were calculated for these six modules separately for domesticated horses (H) and wild equids (P/D/Z). Eigenvalue dispersion values indicated that each of the six modules showed lower magnitudes of integration in domesticated horses (average = 0.68, median = 0.72) compared to wild equids (average = 0.73, median = 0.79). For domesticated horses, eigenvalue dispersion values were lowest for AON (0.53) and highest for MR (0.81) (range = 0.28). For wild equids, integration values were lowest for ZP (0.58) and highest for MR (0.84) (range = 0.26) (Additional file 2: Table S2). The ZP module was most similar in terms of magnitude of integration between domesticated horses and wild equids (difference of 0.03, 4.7%), whereas the CB module had the highest integration in wild equids compared to domesticated horses (difference of 0.07, 9.5%, Additional file 2: Table S2). Module disparity values were higher in domesticated horses compared to wild equids for four out of six modules; these were AON, CV, MR, and ZP (Additional file 2: Table S2). Average disparity across all six modules was the same for both domesticated horses and wild equids (0.034), with disparity ranging from 0.026 (MR) to 0.046 (ZP) for domesticated horses (median = 0.031) and from 0.021 (MR) to 0.041 (ORB) for wild equids (median = 0.035). The ORB module showed the largest difference in disparity between the two groups. Disparity values for the AON module were most similar for domesticated and wild forms (Additional file 2: Table S2). In both wild equids and domesticated horses there is a general trend of increasing disparity with decreasing magnitude of integration. Further, the AON and ZP modules stand out from the other cranial modules as they both show higher disparity coupled with lower integration values (Fig. 2).

## Discussion

The results of our study on skull shape variation support Darwin’s hypothesis that the intraspecific disparity in horses is larger than the interspecific disparity in equids. The shape variation of domesticated horses is not only larger than that of the closest relative (Przewalski’s horses) but similar to the shape variation of all the wild equid species existing today. Horses do not only dominate the Procrustes shape space when comparing crania, but also comparing mandibles or teeth - showing higher shape variation in all tested elements.

**Fig. 3:**
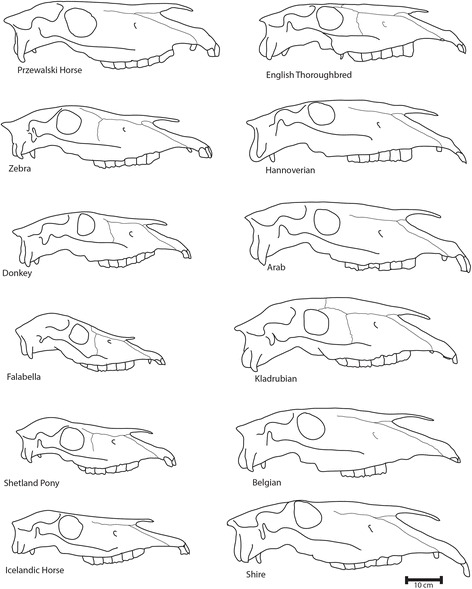
Examples for skull shapes from lateral view of different domesticated (Draft: Shire, Belgian; Light horse: Arab; Medium horses: Hannoverian, Kladrubian, Engl. Thoroughbred; Ponies: Falabella, Shetland, Icelandic) and wild equids (Przewalski’s horse, Zebra, Donkey)

The overall classification among domesticated horses (H), Przewalski’s horses (P), donkeys (D), and zebras (Z) had an average accuracy of 44.5% (range: 34.5% - 78.4%), which lies significantly above the random chance accuracy of 25%. When we excluded the Przewalski’s specimens due to their small sample size the average accuracy increased to 89.2% (range: 63.9% - 98.2%). The separation of caballine from non-caballine taxa and the clustering within these sister clades of H & P, and D & Z by the Mahalanobis distances, is in accordance with the phylogenetic relationship of equids. All four groups descend from a common ancestor around 4–4.5 myr BP, with zebras and donkeys splitting around 2.8 myr BP and Przewalski’s horses and the wild ancestor of today’s domesticated horses splitting 38–72 kyr BP [2, 3, 37]. The first PC of the cranial shape data tended to separate caballine from non-caballine taxa. The accompanying shape differences are dominated by an elongation of the occipital part of the cranium in zebras and donkeys, which previously have been shown to be a distinct character to separate these two taxa from the domesticated horses (for a more detailed morphological description of the skulls of each group see Table 4, Fig. 3, [38, 39]).

**Table 4:** Description of morphological differences for domesticated and wild equids for the studied sample (for a detailed sample composition see Table 2)

Type	Domesticated equids				Wild equids		
	Draft horses (*n* = 37)	Ponies including Falabella (*n* = 40)	Medium horses (*n* = 38)	Light horses (*n* = 14)	Przewalski’s horses (*n* = 5)	Donkeys (*n* = 33)	Zebras (*n* = 47)
Trait							
Average skull length based on studied sample (±SD)	56 (±2.5) cm	43 (±3.6) cm	52 (±4.5) cm	51 (±2.3) cm	46 (±2.1) cm	41 (±4.6) cm	48 (±4.1) cm
General skull shape	Skull breadth is half of skull length	Skull breadth is half of skull length	Elongated	Elongated	Skull breadth is half of skull length	Skull breadth is almost skull length	Very elongated in the Grevy Zebra
Frontals	Broad	Broad in some breeds	Broad in some breeds	Narrow	Narrow	Narrow	Narrow
Degree of cranial flexion (after Bennett, 1980)	High	High in smaller breeds	Medium	High	Medium	High	Low
Nasals	Convex; End at ventral end of premaxillary-maxillary suture	Concave in smallest breed, straight in larger breeds; End before ventral end of premaxillary-maxillary suture	Straight with exceptions in some breeds; End at ventral end of premaxillary-maxillary suture	Concave; End at ventral end of premaxillary-maxillary suture	Straight or slightly concave; End at ventral end of premaxillary-maxillary suture	Straight or slightly concave; End at ventral end of premaxillary-maxillary suture	Straight or slightly concave; End at ventral end of premaxillary-maxillary suture
Angle between premaxilla and maxilla	Obtuse	Obtuse, almost straight	Straight	Straight	Right to obtuse	Obtuse to straight	Obtuse to straight
Facial crest	Prominent	Prominent	Prominent	Less prominent	Prominent	Prominent	Prominent
Zygomatic, temporal and postorbital bars	Very broad, Horizontal to skull midline	Broad to normal; Horizontal to skull midline	Thin, with the temporal being broader; Horizontal to skull midline	Very thin; Horizontal to skull midline	Normal; Angled to skull midline	Normal; Angled to skull midline	Very broad; Angled to skull midline
Orbit shape	Roundish with a more squared part at the dorso-posterior edge	Almost round	Egg-shaped	Oval	Oval	Oval	Round to egg-shaped
Zygomatic process of the temporal bone	Flat	Flat	Curved	Curved	Flat	Flat	Flat
Length of the lambdoidal crest	Does not overlap the condyles in lateral view	Does not overlap the condyles in lateral view	Does not overlap the condyles in lateral view	Does not overlap the condyles in lateral view	Overlaps the condyles in lateral view	Overlaps the condyles in lateral view	Overlaps the condyles in lateral view
Cranial vault	Flat	Rounded in smaller breeds	Flat	Flat	Flat	Rounded	Flat

In contrast to the results of the cranial analysis, we did not find clear group-distinguishing shape differences in the mandible or in any of the investigated teeth. We found the highest Procrustes variance in the mandible data set, very closely followed by the cranium. All four teeth showed a lower Procrustes variance pointing towards less variability, a result also reflected in the low magnitude of disparity for landmarks belonging to the molar (MR) module, probably related to dietary constraints. Our findings on tooth shape differences are congruent with the “long-fuse” hypothesis on teeth by Seetah et al. [14], stating that “shape changes in equids have been modest […] until the development of modern breeds in recent centuries”. Cucchi et al. [16], however, found a strong taxonomic pattern in the shape of the enamel folding, allowing for a more distinct taxonomic separation at the species level. These resulting differences are most likely due to the different choice of teeth ([14]: UP4; [16]: LP3 – LM2), as suggested by one of the articles [16], different wear stages, and/or the different methods used (landmarks vs. semilandmarks).

Modular patterning of the cranium is well-supported by empirical evidence, representing shared development and functional associations between parts of the cranium, resulting in their parcellation into semi-autonomous units. Consistent with patterns recovered for other placental mammals [20], our results indicate that trait variation in the equid cranium is best supported by a six-module hypothesis. These six modules reflect functional groups: the anterior oral-nasal (AON) and molar (MR) modules represents the primary masticatory apparatus, the zygomatic-pterygoid (ZP) module includes the region of attachment for masticatory muscles, the orbit (ORB) module contains the visual sensory organs, the cranial vault (CV) supports and protects the brain, and the cranial base (CB) supports the braincase and is the point of attachment between the skull and axial skeleton. Previous analyses of module disparity and integration for these six modules in a sample of carnivorans provided some support for highly integrated modules showing low disparity, particularly the basicranium (CB), and weakly integrated modules showing high disparity (ORB and ZP) [20, 28]. Our results are broadly consistent with this trend (Fig. 2), which is suggestive of strong integration acting to limit trait variation or the direction of response to selection. The ZP module in horses is also found to be weakly integrated and showing high disparity. Among the carnivoran sample, the molar region showed an unusual pattern of high disparity and high levels of integration [28], in our sample the molar module is also recovered as the most highly integrated module but displays the lowest levels of disparity for both wild and domesticated forms. The discrepancy between these results is likely explained by the diversity of dietary habits represented by the carnivoran sample (e.g. hypercarnivores, insectivores, frugivores) in that earlier study, which resulted in a high disparity among the landmarks captured in the molar module.

Selection acting on shared developmental and functional processes can result in an uncoupling of trait associations at different levels [40–43], providing evidence for complex interactions between modularity and selection. Following, it might therefore be expected that domestication events, as examples of selective breeding regimes, could alter patterns of modularity and integration, and these alterations may differ among breeds, acknowledging that the features targeted for selection (e.g. gait, conformation) are likely to differ for some breeds. There exist few empirical tests of this hypothesis and results on dogs are inconclusive, with reports that patterns of integration have remained stable despite the morphological diversification associated with domestication [9, 44], but also that high module disparity is associated with greater cranial shape variation in dogs compared to wolves [45]. In contrast to Parr et al. [45] our results show highly similar magnitudes of module disparity among wild and domesticated forms, and instead lower magnitudes of module integration are recovered in domesticated horses compared to their wild relatives. Variability in integration magnitude, as recovered here, rather than patterning has been proposed to underlie cranial diversity in mammals [31, 32], such that general conservatism in patterning across mammals may be explained as a product of stabilizing selection on basic developmental processes whereas directional selection could act by altering magnitudes of integration. A recent study conducted simulations to test the role of integration in generating morphological disparity and noted that integration may not affect disparity in morphospace in the way that it is usually measured (as a volume of occupied morphospace or as a measure of dissimilarity), making the relationship between morphospace occupation and modularity results potentially difficult to interpret [46]. That study did not compare shape variation and its partitioning into modules, however our PCA plots indicate that the main axes of shape variation in the equid sample are spread across landmarks located in at least three modules (CV, AON and ZP). Similarly, Parr et al. [45] found shape variance in wild and domesticated dogs to be spread across modules with different magnitudes of integration. It has been suggested that modularity may repartition variance along new directions in morphospace, thereby exploring a greater volume, however the so-far limited empirical evidence appears to raise the question of the extent to which those new directions may be aligned with the axes recovered by eigen-decomposition of shape variables into mathematically orthogonal axes, as happens in ordination techniques such as PCA.

## Methods

A total of 216 crania, 224 mandibles, 198 upper and 141 lower second premolars (U2P and L2P respectively), and 192 upper and 144 lower third molars (U3M and L3M respectively) were analysed (Table 2).

We examined specimens from the following collections: Museum für Naturkunde Berlin (MfN Berlin, Germany), Institut für Haustierkunde (Christian-Albrechts-Universität of Kiel, Germany), Museum für Haustierkunde "Julius Kühn" (University of Halle, Germany), Naturhistorisches Museum Wien (NHW Vienna, Austria), and Museo de la Plata (MLP La Plata, Argentina). The dataset includes all recent species of the genus *Equus* [37]. Due to inconsistent species assignment within zebras and donkeys across museums, we analysed all zebra (cranium *n* = 47; mandible *n* = 48) and donkey (cranium *n* = 31; mandible *n* = 33) species as one group, respectively. We further included five crania and two mandibles of Przewalski’s horses. The largest number of specimens in our data set belongs to the domesticated horses (cranium *n* = 133; mandible *n* = 141) including the following breeds: Ancient Breed (Roman period), Anglo-Norman, Arab, Birkenfelder, Belgian Draft, Bosnian Pony, Clydesdale, Exmoor Pony, Falabella, Galician Farm Horse, Grisons (Graubündner), German Riding Pony, Hannoverian, Hackney, Holstein, Hungarian, Huzule, Icelandic Horse, Indian Pony, Kladrubian, Konik, Kosarian, Lipizzan, Mongolian, Norik, Oldenburgian, Pinzgau, Polish Farm Horse, Scottish Pony, Seneca Sarajevo, Shetland Pony, Shire, Styrian, Suffolk, English Thoroughbred, Togo Pony, Trakehner, and Welsh (Table 2).

**Fig. 4:**
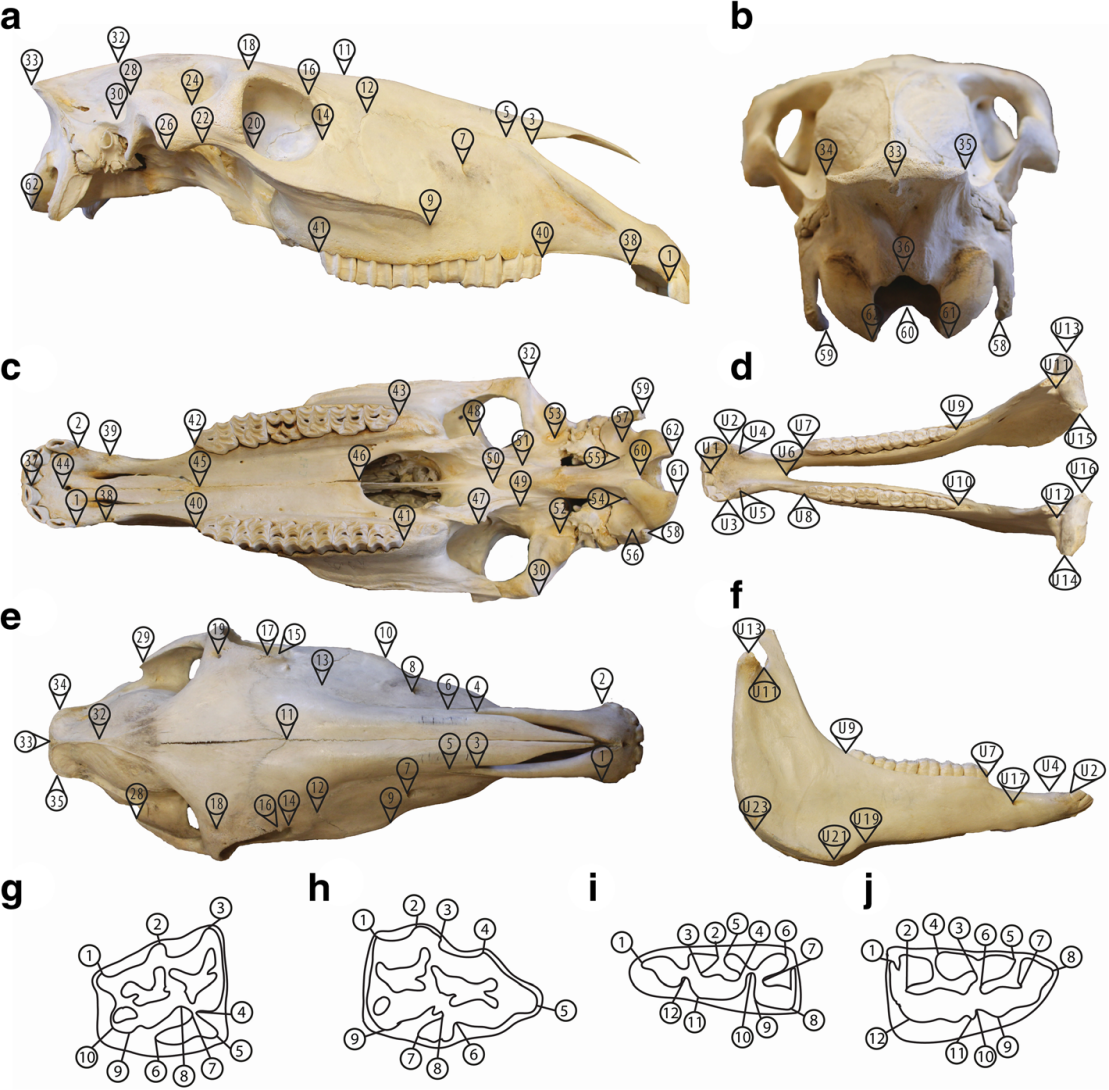
Landmarks on the **a** lateral **b** ventral **c** dorsal **d** posterior side of the skull and the **e** dorsal **f** lateral side of the mandible of a domesticated horse (for a detailed description of the landmarks see Table 3); Landmarks on the **g** upper 3rd molar **h** upper 2nd premolar **i** lower 3rd molar **j** lower 2nd premolar of a zebra (specimen MfN 70,299) in occlusal view (for a detailed description of the landmarks see Table 3)

Analyses of cranial, mandibular and teeth size and shape were performed using landmark-based geometric morphometric (GMM) approaches. The crania and mandibles were measured in three-dimension (3D) using a MicroScribe ® MLX6 (Revware, Inc., Raleigh, North Carolina, USA), while the teeth were measured in two-dimension (2D) using high resolution photographs. A total of 62 type I and type II landmarks [47] were collected on the cranium (Table 5, Additional file 3). The dorsal and ventral sides of the crania (Fig. 4) were measured separately and were subsequently combined using three reference landmarks (numbered 1, 2, and 33, Table 5). For the mandible 24 type II landmark coordinates were measured (Table 5, Fig. 4, Additional file 3).

**Table 5:** Description of the landmarks, including position and type, collected on each cranium, mandible, and tooth; Type I: discrete juxtapositions of tissue types and Type II: maxima of curvature or other local morphogenetic processes [37]

	Position	Type
Cranium		
1–2	Posterior tip of the upper third incisor	II
3–4	Posterior most point of the nasal-premaxilla suture	I
5–6	Premaxillary-maxillary-nasal suture	I
7–8	Dorsoposterior tip of the infraorbital foramen	II
9–10	Anterior tip of the facial crest	II
11	Nasion, nasal-frontal suture, midline	I
12–13	Junction of the lacrimal, maxilla, and nasal sutures	I
14–15	Zygo-lacrimal suture on the orbital margin	I
16–17	Lacrimal-frontal suture on the orbital margin	I
18–19	Supraorbital foramen	II
20–21	Anterior tip of the zygo-temporal suture	I
22–23	Posterior tip of the zygo-temporal suture	I
24–25	Dorsal tip of the frontal-temporal suture	I
26–27	Ventroposterior tip of the zygomatic process	II
28–29	Dorsalmost point of the vertically orientated posterior margin of the zygomatic process	II
30–31	Ventrolateralmost point of squamous part of temporal bone	II
32	Anterior tip of the occipital triangle	I
33	Posterior tip of the nuchal crest	II
34–35	Dorsolateral tip of the nuchal crest	II
36	Dorsalmost point on the margin of the foramen magnum	II
37	Point between first incisors from ventral side	II
38–39	Posteriormost tip of the premaxillary-maxillary suture, ventral	I
40–42	Anterior tip of the second premolar	II
41–43	Posterior tip of the third molar	II
44	Posteriormost point of the incisive canal	II
45	Posterior tip of the palatine process of the incisive bone	I
46	Posterior tip of the palatine-palatine suture	I
47–48	Distal tip of the pterygoid hamulus	II
49–51	Anterior tip of the caudal alar foramen	II
50	Posterior tip of the vomer on the midline	II
52–53	Medial tip of the mandibular fossa	II
54–55	Canal for hypoglossal nerve	II
56–57	Fossa medial of the paracondylar process	II
58–59	Distal tip of the paracondylar process	II
60	Ventral tip of the foramen magnum	II
61–62	Posteriormost tip of the occipital condyle	II
Mandible		
U1	Posterior point between first incisors	II
U2–U3	Posterior tip of the third lower incisor	II
U4–U5	Posterior tip of the canine	II
U6	Posterior tip of the mandible on the midline	II
U7–U8	Anterior tip of the second premolar	II
U9–U10	Posterior tip of the third molar	II
U11–U12	Junction of the bases of the coronoid and condylar processes	II
U13–U14	Lateral tip of the condylar process	II
U15–U16	Medial tip of the condylar process	II
U17–U18	Posterior tip of the mandibular mental foramen	II
U19–U20	Vascular notch of the mandible	II
U21–U22	Maximum curvature of the angle of the mandible right behind the vascular notch	II
U23–U24	Maximum curvature of the angle of the mandible	II
Cranium 2P		
1	Maximum curvature of the metastyle	II
2	Maximum curvature of the mesostyle, distal side	II
3	Maximum curvature of the mesostyle, medial side	II
4	Maximum curvature of the anterior accessory rib	II
5	Maximum curvature of the parastyle	II
6	Maximum curvature of the protocone, mesial/labial side	II
7	Maximum curvature of the protocone, buccal side	II
8	Maximum curvature of the protocone, distal/labial side	II
9	Maximum curvature of the hypocone	II
Cranium 3 M		
1	Maximum curvature of the metastyle	II
2	Maximum curvature of the mesostyle	II
3	Maximum curvature of the parastyle	II
4	Maximum curvature of the preprotoconal groove	II
5	Maximum curvature of the protocone, mesial side	II
6	Maximum curvature of the protocone, distal side	II
7	Maximum curvature of the post protoconal valley, buccal side	II
8	Maximum curvature of the post protoconal valley, labial side	II
9	Maximum curvature of the hypocone, buccal side	II
10	Maximum curvature of the hypocone, labial side	II
Mandible 2P		
1	Maximum curvature of the protoconid, anterior/ lingual side	II
2	Maximum curvature of the preflexid, anterior side	II
3	Maximum curvature of the preflexid, posterior side	II
4	Maximum curvature of the metaconid, anterior side	II
5	Maximum curvature of the metastylid, posterior side	II
6	Maximum curvature of the postflexid, anterior side	II
7	Maximum curvature of the postflexid, posterior side	II
8	Maximum curvature of the hypoconulid, posterior side	II
9	Maximum curvature of the hypoconid, anterior/ buccal side	II
10	Maximum curvature of the ectoflexid, lingual side	II
11	Maximum curvature of the protoconid, posterior/ buccal side	II
12	Maximum curvature of the protoconid, anterior/ buccal side	II
Mandible 3 M		
1	Maximum curvature of the hypoconulid, posterior side	II
2	Maximum curvature of the entoconid, anterior side	II
3	Maximum curvature of the entoflexid, posterior side	II
4	Maximum curvature of the entoflexid, anterior side	II
5	Maximum curvature of the metastylid, posterior side	II
6	Maximum curvature of the metaconid, anterior side	II
7	Maximum curvature of the metaflexid, posterior side	II
8	Maximum curvature of the protoconid, anterior side	II
9	Maximum curvature of the protoconid, posterior side	II
10	Maximum curvature of the ectoflexid, lingual side	II
11	Maximum curvature of the hypoconid, anterior/ buccal side	II
12	Maximum curvature between the hypoconid and hypoconulid, lingual side	II

Phenotypic variation of the four studied teeth was assessed using 9 to 12 two-dimensional landmarks (Type II) (Table 5, Fig. 4, Additional file 4, 5, 6 and 7) following Seetah et al. [48] for the upper teeth that was adapted for the lower teeth. The landmark coordinates were collected on high resolution photographs using TPSDig2 [49]. The photographs were all taken in a standardized manner using a Canon Eos 600d with a Canon EF 24-105 mm f/4 L S USM lens from lateral and dorsal view with a scale bar for size reference.

### Geometric morphometric analyses

To eliminate the effects of size, orientation, and scaling we performed General Procrustes Analysis (GPA, [50]), which translates all specimens’ coordinates so their centroid coincides, scales them to unit centroid size, and rotates them to minimize squared summed distances between matching landmarks. With the cranium and the mandible being symmetric objects, only the symmetric component of shape was analysed in subsequent procedures [51]. The Procrustes scores retained from the GPAs for each skeletal feature were subjected to Principal Component Analyses (PCA). Differences in shape among the four different equid taxa were explored using Procrustes analysis of variance (ANOVA) [52] with shape (PC scores) as the dependent and group (horses [H], Przewalski’s horses [P], zebras [Z], and donkeys [D]) as the independent variable. Canonical Variance Analyses (CVA) was then performed to identify the shape features which best characterize the different groups. Due to the high dimensionality of the datasets, a dimensionality reduction was performed prior to the ANOVA and CVA analyses using the mevolCVP function in R [12]. The mevolCVP function helps to identify the appropriate number of dimensions (first PC scores) which maximize the cross-validated percentage in the subsequent analyses using leave-one-out cross-validated linear discriminant analyses (LDA) (for a more detailed explanation see [53]). We then only used the predetermined number (N) of first PC scores to test for differences in shape among the defined groups using Procrustes ANOVA. If the Procrustes ANOVA showed significant results, we performed a Canonical Variance Analysis (CVA) to identify the shape features which best characterize the different groups. Because sample sizes of Przewalski’s horses were relatively small, CVA analyses were performed with and without them. When they were excluded, predictive CVAs were used to assess the proximity of these specimens with the three remaining groups (identification were based on resampled designs [15]).

We determined distances among the groups by calculating squared Mahalanobis distances (D^2^), which represents the distance of one group mean to another group mean in standard deviations.

Further, we analysed the morphological disparity (as Procrustes variance, [52]) which is the occupied space of all specimens together in multidimensional shape space [54]. First, we calculated the grand mean in unit Procrustes variances. Then we inferred and compared Foote’s partial disparity (PD) [54, 55] to the grand mean. PD was calculated for each group (H, P, D, Z) separately, and for wild equids (D, Z, P) and domesticated horses (H). To do so, the residuals from the regression of shape across all specimens were used and the squared residual lengths were summed over either group mean. The resulting group wise Procrustes variances were multiplied by the number of samples per group divided by total sample size minus one. We then calculated the contribution in percent of each group to the overall disparity.

Analyses were conducted using R [56] in RStudio (v.1.0.136) and related R packages [52, 57, 58] (R script is available upon request). The analyses were computed separately for the cranium, the mandible, and each of the four teeth.

### Modularity analyses

Cranial landmarks for the total sample (wild equids and domesticated horses) were tested for modular structure using 17 models. Wild equids and domesticated horses were pooled because modular patterning has been demonstrated to be stable across placental mammals [20, 31, 32]. With the exception of the simplest model (= no modularity), cranial landmarks were subdivided into modules following a priori hypotheses for modular patterning. These were: 1) Tissue origin hypothesis (neural crest vs paraxial mesoderm derived elements [33]), 2) adult module hypothesis [20], 3) Cheverud’s functional module hypothesis [59], and 4) horse-specific hypothesis [35, 60] (see Additional file 8: Table S3). Hypotheses #1–3 have previously been tested on a macroevolutionary scale in mammals whereas hypothesis #4 tests the face and neurocranium as two separate units based on previously recovered growth pattern differences of the face relative to the neurocranium in horse evolution [35, 36, 60]. For each of these competing hypotheses (#1–4) we compared the fit of our data to different model structures, allowing for variation in correlation within and between modules. As such, each hypothesis was evaluated for four variants (a-d), these were a) same within-module integration and same between-module integration, b) same within-module integration and separate between-module integration, c) separate within-module integration and same between-module integration, and d) separate within-module integration and separate between-module integration (see Additional file 8: Table S3). The fit of the 17 models (4 hypotheses × 4 variants [a-d, above] plus ‘no modularity’ hypothesis) was evaluated using the EMMLi package version 0.0.3 [22] in R, using a coordinate (Procrustes aligned) correlation matrix based on absolute values of correlations as input. EMMLi is a maximum likelihood approach that allows for the direct comparison of models of mixed complexity, and outputs a corrected Akaike Information Criterion (AICc) value and an AICc difference (dAICc), which can be used to assess the fit of the model to the data [22].

The best supported model of modularity (lowest AICc and smallest dAICc) recovered from the EMMLi analysis was chosen for further calculations of module disparity and integration and comparisons between wild and domesticated forms. The cranial landmark matrix was subdivided into matrices for domesticated horses and wild equids. The matrices for domesticated horses and wild equids were each further subdivided into module-specific landmark sets (e.g. orbit module domesticated horses, orbit module wild equids) and subject to GPA. For each module, disparity of the landmarks within that module was defined as maximum Procrustes distance following previous studies (e.g. [45]), and was calculated using Procrustes distances between the mean shape landmark configuration and the landmark configuration of each specimen. Disparity calculations were performed using the Evomorph package version 0.9 [61] in R. For each module, integration of the landmarks within that module was calculated using relative eigenvalue standard deviation (i.e. eigenvalue dispersion), following calculations detailed in [62]. This measure assesses the variance of extracted eigenvalues, which would be maximal when all variation in the data is found in a single dimension (i.e. complete integration) and zero when all eigenvalues are equal (i.e. no integration [30]). Therefore, large values for eigenvalue dispersion reflect strong integration between the landmarks in a module. Eigenvalue dispersion has been shown to be independent of trait number and highly correlated with the mean squared correlation coefficient [32].

## Conclusion

We described and compared shape changes in various skeletal features among extant equid species using geometric morphometrics. Our results support Darwin`s hypothesis that shape variation in the skull of domesticated horses is similar to the shape variation of all wild extant equid species. Our study further shows that lower magnitudes of integration among six cranial modules are found in domesticated horses compared to their wild relatives. Future research could address the relation between integration and disparity, investigating the relation between the two during the domestication process of diverse species.

## Additional files

Additional file 1: **Table S1.** List of cranial landmarks and their placement within the module configurations tested in this study. Four modularity hypotheses were tested, see text for further details. Modules for each hypothesis are as follows; 1. Tissue origin – neural crest (NC), paraxial mesoderm (PM); 2. Mammalian modules – anterior oral-nasal (AON), cranial base (CB), cranial vault (CV), molar (M), orbital (ORB), zygomatic pterygoid (ZP); 3. Functional modules – basicranium (B), frontal (F), masticatory (M), nasal (N), oral (O), orbital (OB); 4. Horse-specific – brain (BR), teeth (TE). (DOCX 15 kb)

Additional file 2: **Table S2.** Results from EMMLi analyses, showing the best (highlighted) supported model of modularity for the cranial landmark data set. Details show the model parameters (K), maximum and log-likelihood values for each tested model, as well as the corrected Akaike Information Criterion (AICc), and the difference between the AICc for a model and the overall minimum AICc (dAICc). The number of between-trait correlations considered in calculating the model likelihood for the sample is 1891, which is equal to the number of unique subdiagonal values of the matrix. Model ID values correspond to those provided in the Material and Methods text. (DOCX 15 kb)

Additional file 3: Raw data for crania and mandibles for all specimens used in this study including three-dimensional landmark data (raw coordinates) and identifier; ID_String is the individual combination including all information: Museum (A = Argentina, B = Berlin, H = Halle, K = Kiel, V = Vienna), ID (identifier used at the museum), group (H = horse, D = donkey, P = Przewalski’s, Z = zebra), breed (aaa = not a domesticated horse, ahb = Ancient Breed (Roman period), ano = Anglo-Norman, arb = Arab, bif = Birkenfelder, blg = Belgian Draft, bos = Bosnian Pony, cds = Clydesdale, exm = Exmoor Pony, fab = Falabella, gbh = Galician Farm Horse, grb = Grisons (Graubündner), grp = German Riding Pony, han = Hannoverian, hny = Hackney, hol = Holstein, hun = Hungarian, huz = Huzule, ice = Icelandic Horse, ind = Indian Pony, kdr = Kladrubian, kon = Konik, kos = Kosarian, lpz = Lipizzan, mon = Mongolian, nor = Norik, odb = Oldenburgian, piz = Pinzgau, pll = Polish Farm Horse, scp = Scottish Pony, ses = Seneca Sarajevo, she = Shetland Pony, shi = Shire, stm = Styrian, suf = Suffolk, tbh = English Thoroughbred, tog = Togo Pony, trk = Trakehner, and wel = Welsh), and morphotype (A = not a domesticated horse, W = medium horse, F = Light horse, C = Draft horse, P = Pony). (XLSX 13 kb)

Additional file 4: TpsDig output for 2) upper third molar, 3) upper second premolar, 4) lower third molar, and 5) lower second premolar for all specimens used in this study including two-dimensional landmark data (raw coordinates) and identifier (image name); for details see Additional file 1. (TXT 13 kb)

Additional file 5: TpsDig output for 2) upper third molar, 3) upper second premolar, 4) lower third molar, and 5) lower second premolar for all specimens used in this study including two-dimensional landmark data (raw coordinates) and identifier (image name); for details see Additional file 1. (TXT 12 kb)

Additional file 6: TpsDig output for 2) upper third molar, 3) upper second premolar, 4) lower third molar, and 5) lower second premolar for all specimens used in this study including two-dimensional landmark data (raw coordinates) and identifier (image name); for details see Additional file 1. (TXT 12 kb)

Additional file 7: TpsDig output for 2) upper third molar, 3) upper second premolar, 4) lower third molar, and 5) lower second premolar for all specimens used in this study including two-dimensional landmark data (raw coordinates) and identifier (image name); for details see Additional file 1. (TXT 12 kb)

Additional file 8: **Table S3.** Module disparity and integration values calculated separately for domesticated and wild horses. Modules are, anterior oral-nasal (AON), cranial base (CB), cranial vault (CV), molar (MR), orbital (ORB), and zygomatic-pterygoid (ZP), as recovered by Goswami (2006) (see Materials and Methods for further details). (DOCX 14 kb)
