# Supplementary material for: Shape variation and modularity of skull and teeth in domesticated horses and wild equids
Source: Front Zool. 2018 Apr 19;15:14. doi: 10.1186/s12983-018-0258-9 (PMC5907714; doi:10.1186/s12983-018-0258-9)
Supplement: Supplementary file 1 — Table S1. List of cranial landmarks and their placement within the module configurations tested in this study. Four modularity hypotheses were tested, see text for further details. Modules for each hypothesis are as follows; 1. Tissue origin – neural crest (NC), paraxial mesoderm (PM); 2. Mammalian modules – anterior oral-nasal (AON), cranial base (CB), cranial vault (CV), molar (M), orbital (ORB), zygomatic pterygoid (ZP); 3. Functional modules – basicranium (B), frontal (F), masticatory (M), nasal (N), oral (O), orbital (OB); 4. Horse-specific – brain (BR), teeth (TE). (DOCX 15 kb) [file 12983_2018_258_MOESM1_ESM.docx]

**Additional file 1: Table S1:** List of cranial landmarks and their placement within the module configurations tested in this study. Four modularity hypotheses were tested, see text for further details. Modules for each hypothesis are as follows; 1. Tissue origin – neural crest (NC), paraxial mesoderm (PM); 2. Mammalian modules – anterior oral-nasal (AON), cranial base (CB), cranial vault (CV), molar (M), orbital (ORB), zygomatic pterygoid (ZP); 3. Functional modules – basicranium (B), frontal (F), masticatory (M), nasal (N), oral (O), orbital (OB); 4. Horse-specific – brain (BR), teeth (TE).

| **Cranial Landmark #** | **Modularity hypothesis** | | | |
| --- | --- | --- | --- | --- |
|  | **1. Tissue origin** | **2. Mammalian modules (Goswami)** | **3. Functional modules (Cheverud)** | **4. Horse-specific**  **(Radinsky)** |
| 1 | NC | AON | O | TE |
| 2 | NC | AON | O | TE |
| 3 | NC | AON | N | TE |
| 4 | NC | AON | N | TE |
| 5 | NC | AON | N | TE |
| 6 | NC | AON | N | TE |
| 7 | NC | AON | N | TE |
| 8 | NC | AON | N | TE |
| 9 | - | - | - | - |
| 10 | - | - | - | - |
| 11 | NC | ORB | N | TE |
| 12 | NC | ORB | N | TE |
| 13 | NC | ORB | N | TE |
| 14 | NC | ORB | OB | - |
| 15 | NC | ORB | OB | - |
| 16 | NC | ORB | OB | - |
| 17 | NC | ORB | OB | - |
| 18 | NC | ORB | F | - |
| 19 | NC | ORB | F | - |
| 20 | NC | ZP | OB | - |
| 21 | NC | ZP | OB | - |
| 22 | NC | ZP | M | - |
| 23 | NC | ZP | M | - |
| 24 | NC | ZP | M | - |
| 25 | NC | ZP | M | - |
| 26 | NC | ZP | M | - |
| 27 | NC | ZP | M | - |
| 28 | NC | ZP | M | - |
| 29 | NC | ZP | M | - |
| 30 | NC | ZP | M | - |
| 31 | NC | ZP | M | - |
| 32 | PM | CV | F | BR |
| 33 | PM | CV | F | BR |
| 34 | PM | CV | F | BR |
| 35 | PM | CV | F | BR |
| 36 | PM | CV | B | BR |
| 37 | NC | AON | O | TE |
| 38 | NC | AON | O | TE |
| 39 | NC | AON | O | TE |
| 40 | NC | AON | O | TE |
| 41 | NC | MR | O | TE |
| 42 | NC | AON | O | TE |
| 43 | NC | MR | O | TE |
| 44 | NC | AON | O | TE |
| 45 | NC | AON | O | TE |
| 46 | NC | MR | O | TE |
| 47 | - | - | - | - |
| 48 | - | - | - | - |
| 49 | NC | ZP | B | BR |
| 50 | NC | ZP | B | BR |
| 51 | NC | ZP | B | BR |
| 52 | NC | ZP | B | BR |
| 53 | NC | ZP | B | BR |
| 54 | PM | CB | B | BR |
| 55 | PM | CB | B | BR |
| 56 | PM | CB | B | BR |
| 57 | PM | CB | B | BR |
| 58 | PM | CB | B | BR |
| 59 | PM | CB | B | BR |
| 60 | PM | CB | B | BR |
| 61 | PM | CB | B | BR |
| 62 | PM | CB | B | BR |
