# Supplementary material for: Shape variation and modularity of skull and teeth in domesticated horses and wild equids
Source: Front Zool. 2018 Apr 19;15:14. doi: 10.1186/s12983-018-0258-9 (PMC5907714; doi:10.1186/s12983-018-0258-9)
Supplement: Supplementary file 2 — Table S2. Results from EMMLi analyses, showing the best (highlighted) supported model of modularity for the cranial landmark data set. Details show the model parameters (K), maximum and log-likelihood values for each tested model, as well as the corrected Akaike Information Criterion (AICc), and the difference between the AICc for a model and the overall minimum AICc (dAICc). The number of between-trait correlations considered in calculating the model likelihood for the sample is 1891, which is equal to the number of unique subdiagonal values of the matrix. Model ID values correspond to those provided in the Material and Methods text. (DOCX 15 kb) [file 12983_2018_258_MOESM2_ESM.docx]

**Additional file 2: Table S2:** Results from EMMLi analyses, showing the best (highlighted) supported model of modularity for the cranial landmark data set. Details show the model parameters (K), maximum and log-likelihood values for each tested model, as well as the corrected Akaike Information Criterion (AICc), and the difference between the AICc for a model and the overall minimum AICc (dAICc). The number of between-trait correlations considered in calculating the model likelihood for the sample is 1891, which is equal to the number of unique subdiagonal values of the matrix. Model ID values correspond to those provided in the Material and Methods text.

| **Model ID** | **Description** | **Max. Likelihood** | **K** | **AICc** | **dAICc** | **Model**  **Likelihood** | **Posterior probability** |
| --- | --- | --- | --- | --- | --- | --- | --- |
| 0 | No.modules.default | -1803.56 | 2 | 3611.131 | 4580.472 | 0 | 0 |
| 1a | Tissue.same.Mod + same.between | -1737.9 | 4 | 3483.825 | 4453.167 | 0 | 0 |
| 1b | Tissue.same.Mod + sep.between | -1737.9 | 4 | 3483.825 | 4453.167 | 0 | 0 |
| 1c | Tissue.sep.Mod + same.between | -1056.95 | 5 | 2123.929 | 3093.27 | 0 | 0 |
| 1d | Tissue.sep.Mod + sep.between | -1056.95 | 5 | 2123.929 | 3093.27 | 0 | 0 |
| 2a | Goswami.same.Mod + same.between | -877.315 | 4 | 1762.651 | 2731.993 | 0 | 0 |
| 2b | Goswami.same.Mod + sep.between | -313.031 | 18 | 662.4267 | 1631.768 | 0 | 0 |
| *2c* | *Goswami.sep.Mod + same.between* | *-56.3181* | *9* | *130.7318* | *1100.073* | *1.32E-239* | *1.32E-239* |
| 2d | Goswami.sep.Mod + sep.between | 507.9663 | 23 | -969.341 | 0 | 1 | 1 |
| 3a | Functional.same.Mod + same.between | -1085.46 | 4 | 2178.933 | 3148.274 | 0 | 0 |
| 3b | Functional.same.Mod + sep.between | -487.41 | 18 | 1011.185 | 1980.527 | 0 | 0 |
| 3c | Functional.sep.Mod + same.between | -945.554 | 9 | 1909.204 | 2878.545 | 0 | 0 |
| 3d | Functional.sep.Mod + sep.between | -347.508 | 23 | 741.6077 | 1710.949 | 0 | 0 |
| 4a | horse.same.Mod + same.between | -1218.03 | 4 | 2444.088 | 3413.429 | 0 | 0 |
| 4b | horse.same.Mod + sep.between | -1218.03 | 4 | 2444.088 | 3413.429 | 0 | 0 |
| 4c | horse.sep.Mod + same.between | -1036.53 | 5 | 2083.093 | 3052.434 | 0 | 0 |
| 4d | horse.sep.Mod + sep.between | -1036.53 | 5 | 2083.093 | 3052.434 | 0 | 0 |
