# Supplementary material for: Shape variation and modularity of skull and teeth in domesticated horses and wild equids
Source: Front Zool. 2018 Apr 19;15:14. doi: 10.1186/s12983-018-0258-9 (PMC5907714; doi:10.1186/s12983-018-0258-9)
Supplement: Supplementary file 8 — Table S3. Module disparity and integration values calculated separately for domesticated and wild horses. Modules are, anterior oral-nasal (AON), cranial base (CB), cranial vault (CV), molar (MR), orbital (ORB), and zygomatic-pterygoid (ZP), as recovered by Goswami (2006) (see Materials and Methods for further details). (DOCX 14 kb) [file 12983_2018_258_MOESM8_ESM.docx]

**Additional file 8: Table S3:** Module disparity and integration values calculated separately for domesticated and wild horses. Modules are, anterior oral-nasal (AON), cranial base (CB), cranial vault (CV), molar (MR), orbital (ORB), and zygomatic-pterygoid (ZP), as recovered by Goswami (2006) (see Materials and Methods for further details).

| **Module** | **Integration (Eigenvalue dispersion)** | | | **Disparity (Procrustes distance)** | |
| --- | --- | --- | --- | --- | --- |
|  | **Domesticated** | **Wild** | **Domesticated** | | **Wild** |
| AON | 0.532187 | 0.59987 | 0.043257 | | 0.04116 |
| CB | 0.711104 | 0.785558 | 0.026985 | | 0.029178 |
| CV | 0.740895 | 0.785045 | 0.033325 | | 0.030771 |
| MR | 0.807072 | 0.840303 | 0.026379 | | 0.021098 |
| ORB | 0.72742 | 0.798126 | 0.028563 | | 0.041428 |
| ZP | 0.554181 | 0.581242 | 0.046471 | | 0.039242 |
| average | 0.678809 | 0.731690 | 0.034163 | | 0.033812 |
